# Generation of lung epithelial-like tissue from human embryonic stem cells

**DOI:** 10.1186/1465-9921-10-105

**Published:** 2009-11-05

**Authors:** Lindsey Van Haute, Gert De Block, Inge Liebaers, Karen Sermon, Martine De Rycke

**Affiliations:** 1 Department of Embryology and Genetics, UZ Brussel, Belgium; 2 Vrije Universiteit Brussel and Centre for Medical Genetics, UZ Brussel, Belgium

## Abstract

**Background:**

Human embryonic stem cells (hESC) have the capacity to differentiate *in vivo *and *in vitro *into cells from all three germ lineages. The aim of the present study was to investigate the effect of specific culture conditions on the differentiation of hESC into lung epithelial cells.

**Methods:**

Undifferentiated hESC, grown on a porous membrane in hESC medium for four days, were switched to a differentiation medium for four days; this was followed by culture in air-liquid interface conditions during another 20 days. Expression of several lung markers was measured by immunohistochemistry and by quantitative real-time RT-PCR at four different time points throughout the differentiation and compared to appropriate controls.

**Results:**

Expression of *CC16 *and *NKX2.1 *showed a 1,000- and 10,000- fold increase at day 10 of differentiation. Other lung markers such as *SP-C *and *Aquaporin 5 *had the highest expression after twenty days of culture, as well as two markers for ciliated cells, *FOXJ1 *and *β-tubulin IV*. The results from qRT-PCR were confirmed by immunohistochemistry on paraffin-embedded samples. Antibodies against CC16, SP-A and SP-C were chosen as specific markers for Clara Cells and alveolar type II cells. The functionality was tested by measuring the secretion of CC16 in the medium using an enzyme immunoassay.

**Conclusion:**

These results suggest that by using our novel culture protocol hESC can be differentiated into the major cell types of lung epithelial tissue.

## Background

Human embryonic stem cells (hESC) [1] have the capacity to differentiate *in vivo *and *in vitro *into cells from all three germ lineages [2], which makes them very interesting in developmental biology, regenerative medicine and *in vitro *pharmacological studies. Furthermore, the derivation of hESC lines carrying a monogenic disease offers the opportunity of creating an *in vitro *model of the disease. Such lines can be derived from an embryo diagnosed as affected after preimplantation genetic diagnosis, a procedure that allows the detection of a genetic defect at the preimplantation embryo level [3]. The *in vitro *generation of lung cells and tissues from human embryonic stem cells provides an alternative to lung transplantation in patients with lung injury due to chronic pulmonary disease and inherited genetic diseases such as cystic fibrosis (CF).

The respiratory system originates from the foregut endoderm, differentiating into many kinds of specialized epithelial cells. These include ciliated, secretory, and neuroendocrine cells of the proximal bronchi, and the alveolar cells. The latter can be divided into two types of cells; type I cells, with a highly flattened morphology ideally suited for gas exchange, and the less differentiated, more cuboidal type II cells serving as progenitor cells for type I cells. Furthermore, these alveolar type II cells are important in synthesizing and secreting pulmonary surfactant proteins, a complex mixture of proteins and phospholipids which lower surface tension [4]. One of these proteins, i.e. SP-C, is expressed in the distal epithelium during early lung development, and becomes restricted to alveolar type II cells late in gestation and postnatally.

Nk2 homeobox1 (NKX2.1) (also Thyroid transcription factor 1 (TTF-1)) is the earliest known marker associated with commitment of endodermal cells to pulmonary and thyroid cell lineages; it appears before the definitive lung formation [5].

During fetal lung development the level of NKX2.1 decreases with advancing age. At first it is expressed in all pulmonary epithelial cells, later it becomes restricted to distal alveolar type II cells and proximal Clara Cells - the nonciliated, nonmucous cells lining the bronchioles of the lung [6]. It is known that these latter cells secrete a variety of proteins, such as the surfactant proteins SP-A, SP-B and SP-D, and most importantly CC16, a Clara cell diffusible 16 kDa protein (previously known as CC10), which appears to be secreted constitutively at a high level throughout life [7].

Mucociliary clearance is an essential aspect of the innate defence mechanisms of the lung. Mucus secreting cells act in concert with the ciliated cells within the pulmonary epithelium to remove potentially harmful substances from the lung. FOXJ1 is a transcription factor expressed in the ciliated cells of the lung and reproductive tracts. It functions in late-stage ciliogenesis of lung development and is expressed before the appearance of ciliated cells [8]. Many genes expressed in the lung are also expressed in other tissues, such as thymus and pancreas. Lung epithelial-like tissue arising within a complex culture can therefore only be identified by measuring the gene expression of multiple markers.

Few research groups have reported on the successful differentiation towards endodermal lineages such as lung epithelium.

We have adapted the approach of Coraux et al. culturing murine ESC in air liquid interface (ALI) conditions, which results in a simple and reliable protocol to differentiate human ESC into a lung epithelial-like tissue. This is to our knowledge the first study to report the differentiation of human ESC into a lung epithelial-like tissue.

## Materials and methods

### hESC Cultures and differentiation

#### Maintenance of hESC

hESC were derived, as previously described [13], from human preimplantation embryos after IVF or preimplantation genetic diagnosis, characterised according to the International Stem Cell Initiative guidelines and karyotyped by array-based comparative genomic hybridization (array-CGH) [14]. The inner cell masses of human blastocysts were obtained by immunosurgery and transferred to 0.1% gelatine-coated culture dishes containing mitomycin C inactivated CF1 mouse embryonic fibroblast feeders (MEF). Upon derivation, cells were routinely cultured on MEF feeders in a humidified atmosphere at 10% CO2 at 37°C in hESC medium that consisted of 80% knockout Dulbecco's modified Eagle's medium (KO-DMEM) supplemented with 20% knockout-serum replacement (KO-SR), 2 mM L-glutamine, 1% nonessential amino acids, 4 ng/ml human recombinant basic fibroblast growth factor (bFGF) and Penicillin/streptomycin (100 U/mL) (all Invitrogen, Carlsbad, CA, USA), and 0.1 mM β-mercaptoethanol (Sigma-Aldrich, St. Louis, MO, USA). Medium was changed daily and passaging was done by mechanical slicing. Five different cell lines were used to check for possible variations in their differentiation potential: VUB03_DM1 carrying a mutation for myotonic dystrophy, VUB04_CF carrying one mutation in the *CFTR *gene, VUB09_FSHD carrying a mutation for facioscapulohumeral muscular dystrophy, and VUB07 and VUB14, neither carrying any known genetic disease.

The lines were at passages 78-80, 44-47, 73-75, 110-112 and 23-25 respectively, when used for our experiments. The main characteristics of the cell lines, including the chromosomal abnormalities detected by array-CGH, are summarized in additional file 1.

#### Differentiation of hESC

Collagenase IV dissociated hESC clumps of variable sizes (± 30/insert) were plated on porous membranes of millicell-HA culture inserts (Millipore Corporation, Billerica, MA, USA). These culture inserts were placed in 12-well plates coated with MEF feeders and allowed to expand for four days in hESC medium in liquid-liquid conditions. Differentiation was induced by replacing the medium with differentiation medium (hESC medium without bFGF and β-mercaptoethanol) in liquid-liquid conditions for four days and then switching to air-liquid interface culture for twenty days, in which the medium from the upper compartment was removed (this will be referred to as the "ALI differentiation protocol"). Medium was changed daily. Two types of controls were used in the quantitative real-time RT-PCR experiments: 1) hESC plated on 0.1% gelatine-coated culture dishes on MEF feeders that had differentiated spontaneously in hESC medium (named "hESC spontaneous differentiation control") and 2) hESC plated on porous membranes for four days in hESC medium followed by 24 days in differentiation medium in liquid-liquid conditions (named "NO ALI control"). A third type of control, namely hESC grown on porous membranes in liquid-liquid conditions in hESC medium for two days and referred as "undifferentiated hESC control", was used in the immunostaining experiments.

### RNA extraction and quantitative real-time RT-PCR analysis

After removal of the cells from the porous membrane with lysis buffer from the RNeasy kit, RNA was extracted and treated with RNase-free DNase according to the manufacturer's protocol (both Qiagen, Hilden, Germany). Five micrograms of total RNA were reverse transcribed into cDNA using the First Strand cDNA Synthesis Kit (GE Healthcare, Buckinghamshire, UK) with the NotI-d(T)18 primer, according to the manufacturer's instructions.

PCR reaction mixtures contained cDNA template, 12,5 μL 2× Eurogentec qPCR Mastermix (Eurogentec, Liege, Belgium) in a final volume of 25 μL. Primers and probes were added either as a mix (1,25 μL 20× TaqMan Gene expression assay (Applied Biosystems, Foster City, CA, USA) or separately (900 nM and 250 nM respectively). Primers and probes were designed using PrimerExpress software (Applied Biosystems). Primer (Eurogentec) and probe (Applied Biosystems) sequences and amplicon length as shown in table 1. All probes were MGB probes labelled with FAM. Regarding *POU5F1 *(or *OCT4*) it should be noted that we designed our primers and probes not to detect *OCT4B*, since this could lead to false positive results [15].

**Table 1:** Real-time PCR primers and probes

Gene	Primers (5' to 3')	Probe	Amplicon length
GAPDH	ATG-GAA-ATC-CCA-TCA-CCA-TCT-T	CAG-GAG-CGA-GAT-CC	57
	CGC-CCC-ACT-TGA-TTT-TGG		

UBC	CGC-AGC-CGG-GAT-TTG	TCG-CAG-TTC-TTG-TTT-GTG	58
	TCA-AGT-GAC-GAT-CAC-AGC-GA		

FOXJ1	GAT-CAC-GGA-CAA-CTT-CTG-CTA-CTT-C	CCA-CGC-AGA-TCC-CA	74
	GAG-ACA-GGT-TGT-GGC-GGA-TT		

SP-C	GTC-TCC-ACA-TGA-GCC-AGA-AAC-A	ACG-GAG-ATG-GTT-CTG-G	57
	GCG-CCC-CAA-TGC-TCA-TC		

CC16	CCC-TGG-TCA-CAC-TGG-CTC-TC	TGC-AGC-TCC-GCT-TC	61
	TGA-AAG-CTC-GGG-CAG-ATC-TC		

Â-Tubulin IV	GAT-CTT-TCG-GCC-GGA-CAA	TTG-GCC-AAT-CCG-G	65
	GCC-CCT-TTG-CCC-AGT-TG		

NKX2.1	CAC-ACG-ACT-CCG-TTC-TCA-GTG-T	TGA-CAT-CTT-GAG-TCC-CCT-G	66
	GCC-CAC-TTT-CTT-GTA-GCT-TTC-C		

POU5F1	GGA-CAC-CTG-GTC-TGC-GAT-TT	GCC-TTC-TCG-CCC-CC	54
	CAT-CAC-CTC-CAC-CAC-CTG-G		

Aq5	Hs00387048_m1*		68

Vimentin	Hs00185584_m1*		73

Nanog	Hs02387400_g1*		109

* Commercially obtained. Not designed in-house

PCR reactions were performed on an ABI 7500 thermal cycler (Applied Biosystems). Thermal cycling parameters were: 2 min at 50°C, 10 min at 95°C, followed by 40 cycles of 15 s at 95°C and 1 min at 60°C. Measurements were taken at the end of the extension phase at 60°C.

Relative quantification of gene expression between multiple samples was achieved by normalization against two endogenous controls *GAPDH *and *Ubiquitin C *(as recommended by Willems et al. [16]) using the ΔΔCt method of quantification. Relative amount of mRNA was calculated as 2^-ΔΔCt ^and quantitative RT-PCR results were statistically analysed by one-way ANOVA.

Experiments were performed at least three times, and at least two different samples were taken for each measurement to provide biological replicates. Experiments were repeated on four different cell lines (VUB03_DM1, VUB04_CF, VUB07 and VUB14). Each PCR reaction was performed in duplicate as a technical replicate. No-template negative controls were included. Samples were compared to hESC spontaneous differentiation control samples and/or NO ALI control samples. Primers and probes were designed to avoid murine mRNA amplification. Nevertheless, to exclude possible contamination with murine mRNA from MEF, samples with cDNA derived from mouse fibroblasts were included in the different assays. No signal could be measured by quantitative real-time RT-PCR, except for *Vimentin*.

### Immunohistochemistry

#### Immunofluorescence staining

hESC grown on porous membranes following the ALI differentiation protocol were fixed in 3.7% formaldehyde (Merck, Darmstadt, Germany) when still on the membrane, dehydrated and embedded as such in paraffin. For SP-A staining, tissue was embedded in a perpendicular direction as well. Undifferentiated hESC control samples were treated in the same way as a control. Sections (4 μm thick) were rehydrated in gradual stages. For SSEA4, TRA1-60 and TRA 1-81, an additional step with antigen retrieval was performed by heating at 95° for 75 min in freshly made 0.01 M citrate buffer, pH 6.0. Non-specific binding was blocked for 2 h with PBS containing 0.25% Triton-X (Sigma Aldrich) and 2% of the appropriate normal serum (Sigma Aldrich).

Sections were incubated with goat polyclonal antibody to CC16 (Santa Cruz Biotechnology, Santa Cruz, CA; dilution 1/100), goat anti-human polyclonal antibody to SP-C (Santa Cruz: dilution 1/100), SP-A (Chemicon, Temecula, CA; dilution 1/500), SSEA4 (Millipore Corporation, Billerica, MA, USA), dilution 1/100), TRA1-60 (Millipore; dilution 1/100) or TRA1-81 (Millipore; dilution 1/100) overnight at 4°C. After washing for 30 min in PBS, sections were incubated with the corresponding Alexa 488/633 conjugated secondary antibodies for 2 h. After washing in PBS, sections were mounted with Prolong^® ^Gold Antifade reagent with DAPI (all Invitrogen)

Double stainings were performed by sequentially adding the two sets of primary and secondary antibodies.

#### Immunostaining for light microscopy

Immunostaining for Vimentin was performed on both sections of fetal lung tissue (positive control) and hESC grown on porous membranes following the ALI differentiation protocol. Hydrogen peroxide (0.3%) was used to block endogenous peroxidase (15 min). The staining required antigen retrieval (95°C for 75 min in freshly prepared 0.01 M citrate buffer, pH 6.0). Vimentin monoclonal mAb (Dako, Denmark) was used (dilution 1/200) and incubated overnight at 4°. The day after the HRP-labelled secondary antibody was applied for 1 h and staining was finalised with a diaminobenzidine solution (Dako REAL Envision Detection Systems Peroxidase/DAB Rabbit/Mouse, Dako, Denmark) so as to detect the sought after antigen. Slides were extensively washed with PBS between the different stages and counterstained with hematoxylin before mounting.

Both for fluorescence and light microscopy immunostainings, fetal lung and kidney tissue (from a 22- week old fetus) were included as a positive and negative control respectively. Other negative controls were performed by omitting the primary antibodies or replacing them with antibody isotype controls from the appropriate host species at the same concentration.

### Enzyme Immunoassay

Analysis of CC16 protein secretions in the medium was performed using a CC16 Elisa Kit (DiaMed Eurogen, Turnhout, Belgium) according to the manufacturer's instructions and the absorbancy values at 450 nm were determined. The minimum detectable concentration was <50 pg/mL (as defined by the manufacturer). Medium was collected after 24 h and proteins were concentrated by centrifugation for 45 min on 4000 rpm in a Amplicon Ultra-15 Centrifugal filter unit with Ultracel-3 membranes (Millipore). Experiments were performed on three different cell lines (VUB04_CF, VUB07 and VUB09_FSHD).

Standard sera samples were used to generate a standard curve, from which the concentration of CC16 in the samples was interpolated.

Differentiation medium that had not been in contact with any cells was used as a negative control.

## Results

### Characterising the differentiated cells by quantitative real-time RT-PCR

**Figure 1:**
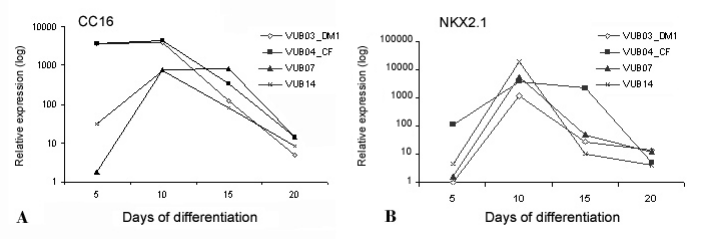
**Relative quantification of *CC16 *and *NKX2.1 *mRNA levels in four different hESC lines at day 5, 10, 15 and 20 of ALI culture**. Relative quantification of *CC16 *(A) and *NKX2.1 *(B) mRNA levels in samples from human embryonic stem cells (hESC) cultured on porous membranes according to the ALI differentiation protocol. Samples were collected on days 5, 10, 15, and 20 of ALI culture. A peak expression is seen at day 10, with a 1000 and 10000- fold expression, followed by a decrease further over time. The NO ALI control samples were considered as reference. (Note that the y-axis is on a logarithmic scale). Two endogenous controls were used (GAPDH and Ubiquitin C). These experiments were repeated at least three times with VUB03_DM1, VUB04_CF, VUB07 and VUB14 hESC lines and data are presented here as means.

**Figure 2:**
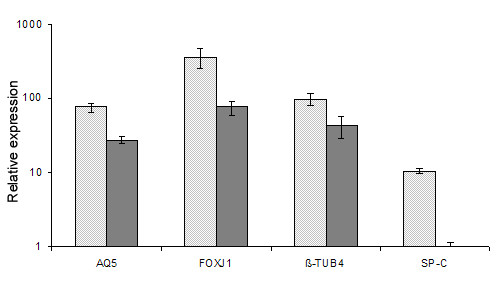
**Quantitative RT-PCR analysis of *Aquaporin 5*, *FOXJ1, β-tubulin IV *and *SP-C *mRNA levels at day 20**. *Aquaporin 5*, *FOXJ1*, *β-tubulin IV *and *SP-C *mRNA levels were measured in samples from hESC cultured on porous membranes with the ALI differentiation protocol and in NO ALI control samples. After 20 days of ALI culture Aquaporin 5, *FOXJ1 *and *β-tubulin IV *are all significantly upregulated (p < 0.05) as is *SP-C *compared to NO ALI control samples. Note that the y-axis is on a logarithmic scale. The samples from hESC spontaneous differentiation control samples were considered as a reference and data represent the mean ± SD. Experiments were performed at least three times and at least two different samples were taken for each measurement to provide biological replicates. Data are shown for VUB07. These experiments were also carried out with VUB03_DM1, VUB04_CF and VUB14 and showed similar results (data not shown). Two endogenous controls were used (*GAPDH *and *Ubiquitin C*).

The differentiation of hESC following the ALI differentiation protocol into different airway epithelial cells was assessed by relative quantitative real-time RT-PCR of lung marker expression. The mRNA levels of *CC16*, and *NKX2.1 *were studied at different time intervals (5, 10, 15 and 20 days of ALI culture) (Figure 1). *CC16 *expression, which is specific for Clara cells and could not be detected in hESC spontaneous differentiation control samples (data not shown), peaked at day 10 (1000-fold increase) and then gradually decreased to about 10-fold at day 20 of ALI culture for all four cell lines tested. The early marker *NKX2.1 *showed a similar pattern: it was not expressed in hESC spontaneous differentiation control samples (data not shown) and was strongly upregulated at day 10 (10000-fold) of ALI culture. At day 5 of ALI culture, the mRNA levels of both genes varied substantially in the four cell lines, but the overall pattern over time was quite similar. The upregulation versus the NO ALI control samples (set as reference) is significant for both *CC16 *and *NKX2.1 *(Figure 1). The mRNA levels of *SP-C, Aquaporin 5, β-tubulin IV and FOXJ1 *were studied at even time intervals (5, 10, 15 and 20 days of ALI culture), but only data at day 20 are presented since expression levels at earlier time points remained low or undetectable (Figure 2). The mRNA levels for *SP-C*, an early distal lung epithelial marker, and for *Aquaporin-5*, coding for a water channel protein and in lungs only expressed in alveolar type I cells, were upregulated 10- and 100 -fold at day 20 of ALI culture compared to hESC spontaneous differentiation control samples (set as reference). The upregulation of *SP-C *and *Aquaporin 5 *is statistically significant compared to the reference samples and to NO ALI control samples as well (Figure 2). At day 20, two markers for ciliated cells, *FOXJ1 *and *β- tubulin IV*, are upregulated more than 100-fold, which is a significant elevation (p < 0.05) compared to both the reference samples (hESC spontaneous differentiation control samples) and the NO ALI control samples.

**Figure 3:**
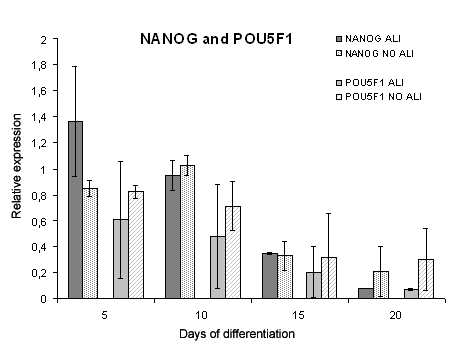
**Relative quantification of *NANOG *and *POU5F1 *mRNA levels**. Relative quantification of *NANOG *and *POU5F1 *mRNA levels in samples from human embryonic stem cells (hESC) cultured on porous membranes with the ALI differentiation protocol (ALI), compared to the NO ALI control samples. Samples were collected on days 5, 10, 15, and 20, and data are represented as means ± SD. *NANOG *and *POU5F1 *mRNA levels decreased over time in both conditions and no statistical difference could be observed. Two endogenous controls were used (*GAPDH *and *Ubiquitin C*). Results of VUB04_CF are shown.

**Figure 4:**
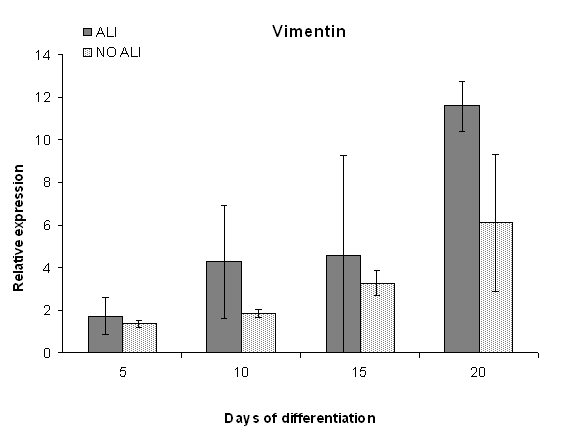
**Quantitative RT-PCR analysis of Vimentin**. Relative mRNA quantification of *Vimentin *in samples from human embryonic stem cells (hESC) cultured on porous membranes with the ALI differentiation protocol compared to the NO ALI control samples. Samples were collected on days 5, 10, 15, and 20, and data are represented as means ± SD. In both conditions an increase in expression levels could be observed. Control cultures showed a slighter increase in expression level, though no statistical difference could be measured. Results of VUB04_CF are shown.

Differentiation involves the downregulation of pluripotency markers. Therefore the mRNA levels of *NANOG *and *POU5F1 *were assessed (Figure 3). *NANOG *and *POU5F1 *mRNA expression decreased over time in both hESC samples cultured with the ALI differentiation protocol and in NO ALI control samples. No significant difference was found between these two conditions. The mRNA expression of *Vimentin*, a marker for mesenchymal cells, increased over time in both conditions, although ALI conditions showed a stronger increase at all time points measured (Figure 4).

The control samples containing only MEF were found to be positive for *Vimentin *as well. Potential contamination with murine mRNA in samples would be similar and would not lead to an underestimation or an overestimation of the *Vimentin *mRNA level in ALI conditions versus NO ALI control samples.

### Characterising the differentiated cells by immunofluorescence staining

During differentiation, cells became covered with a tough mucus-like layer, which dissolved during fixation. On sections, bronchiolar-like structures were seen within the cell clumps.

**Figure 5:**
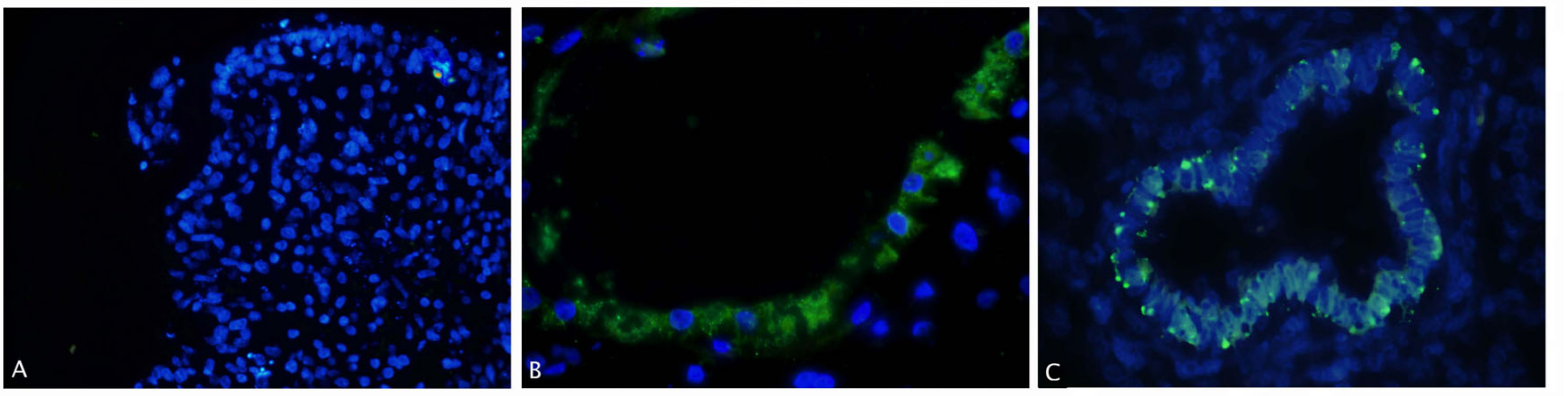
**Immunostaining of CC16**. Representative image of CC16 immunostaining (green) in combination with nuclear DAPI staining (blue) in undifferentiated hESC control samples (A), in samples cultured with the ALI differentiation protocol (VUB03_DM1)(B) and in fetal lung (20 weeks old)(C). Staining was found in the cytoplasm of the cells lining the bronchiolar epithelium (C) and in the cells lining the cavities (B) of the tissue formed by ALI culture. No staining was seen in undifferentiated hESC controls (A). Original magnification 200× (A) and 400× (B-C) These experiments were also carried out with VUB04_CF and with VUB09_FSHD hESC lines and showed similar results (data not shown).

To better characterise the different cells of the ALI cultures, immunofluorescence staining was performed for CC16, a Clara Cell marker and SP-C, known to be exclusively produced in alveolar type II cells. In addition, SP-A, a more mature marker for both cell types, was also stained. When hESC were cultured with the ALI differentiation protocol, CC16 expression could clearly be seen in the cytoplasm of the cells lining the cavities of the tissue (Figure 5A-C).

**Figure 6:**
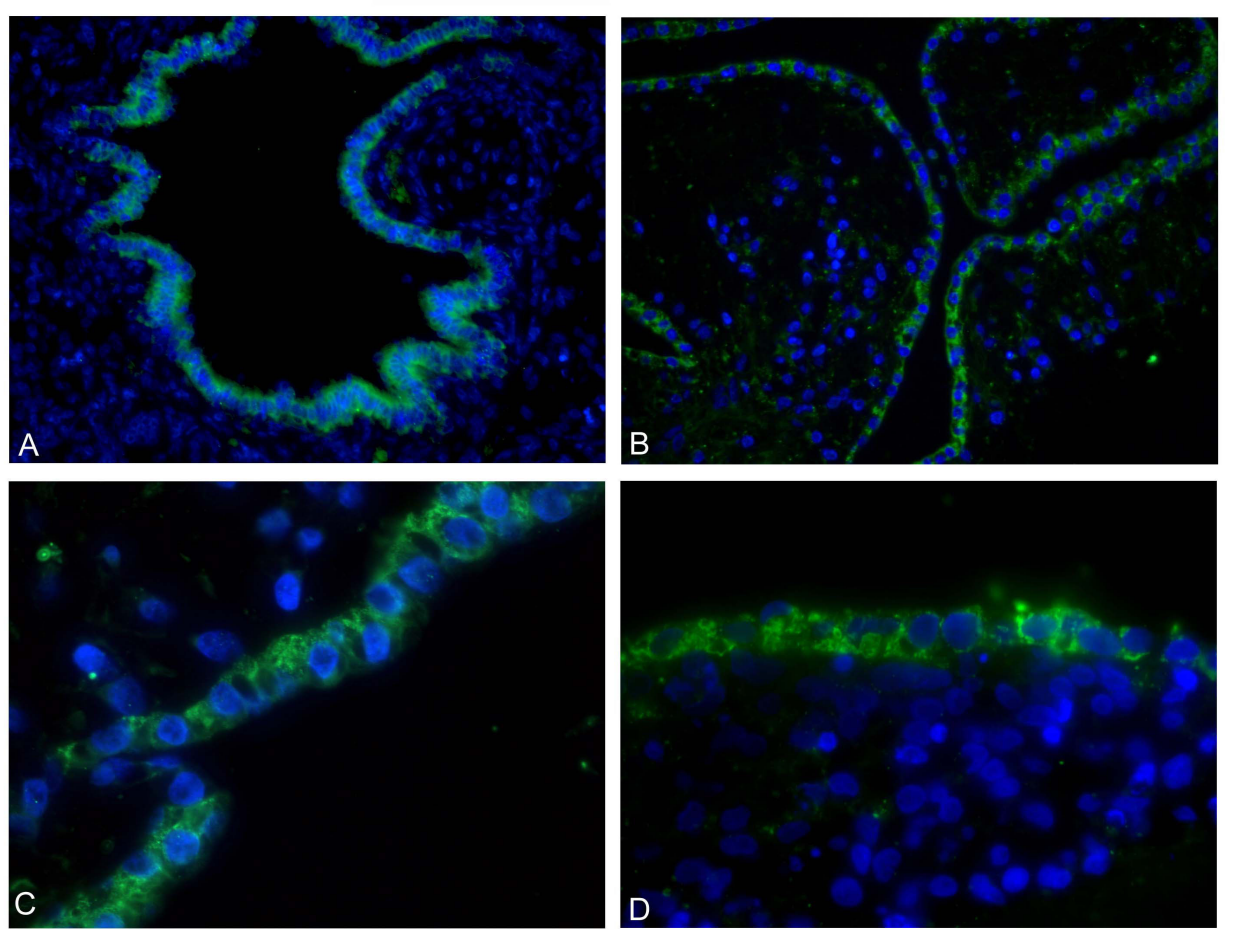
**Immunostaining of SP-C**. hESC were grown with the ALI differentiation protocol for 20 days (B-D). Images of two different cell lines (VUB03_DM1 (B-C) and VUB04_CF (D) were scanned by confocal microscopy after immunostaining with SP-C antibody (green) and nuclear DAPI staining (blue). Figure 6A shows immunostaining of SP-C in fetal lung (20 weeks old). Original magnification 200× (A,B) and 600× (C-D).

**Figure 7:**
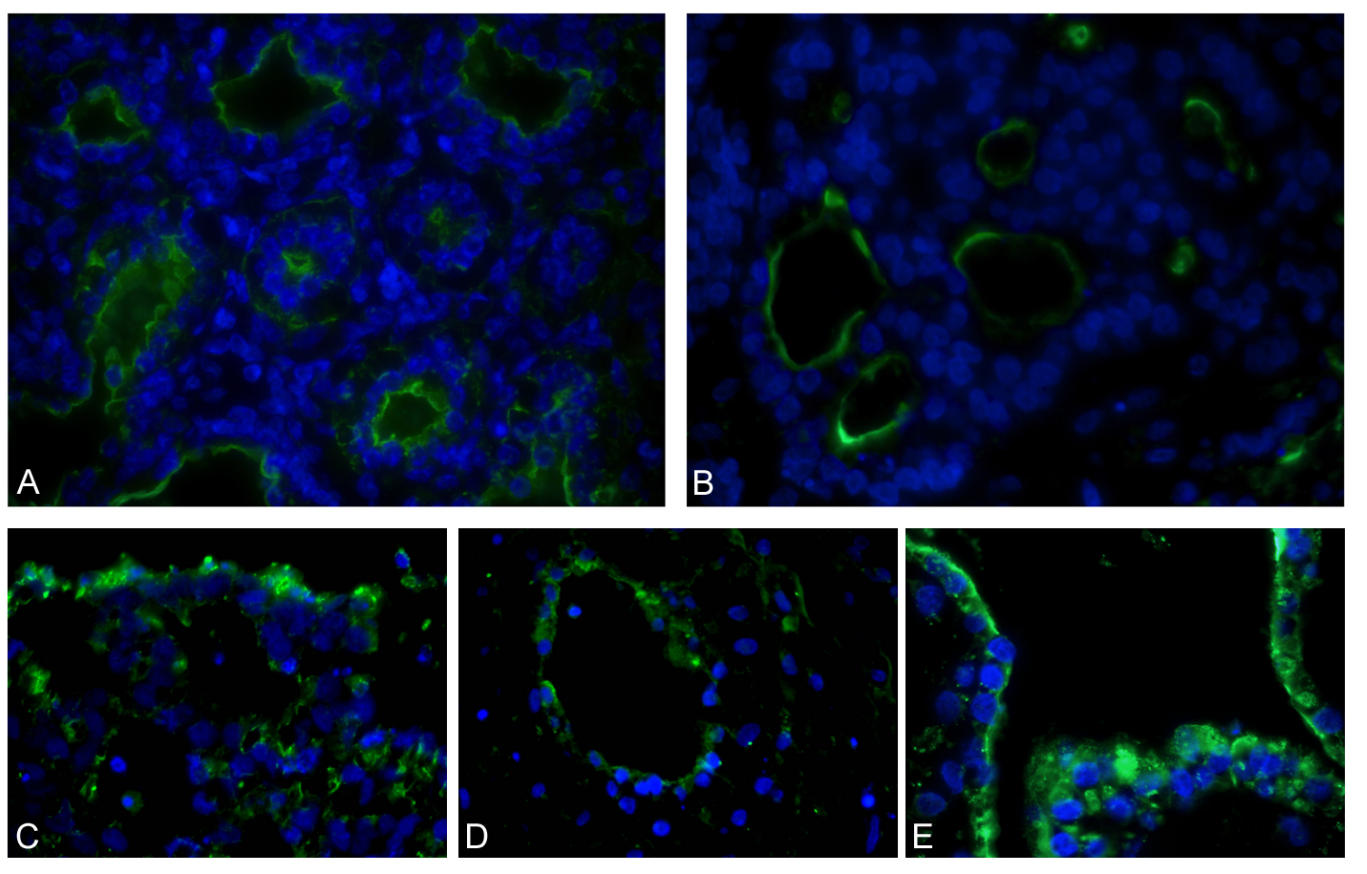
**Immunostaining of SP-A**. Fluorescence microscopic pictures of sections immunostained with SP-A antibody (green) and nuclear DAPI staining (blue). For fetal lung tissue (22 weeks old) (magnification 200×) (A) and hESC cultured with the ALI differentiation protocol at day 20 (VUB03_DM1 (B (magnification 400×) Pictures of sections in a perpendicular direction are shown on three different cell lines: VUB07 (C), VUB03_DM1 (D) and VUB04_CF (E).

Immunostaining analysis also demonstrated SP-C, specific for alveolar type II cells, in the cytoplasm of the cells near the periphery of the cell clumps (Figure 6). No SP-C staining was found in the nuclei of the cells. SP-A stained positively on the edge of the bronchiolar-like structures (Figure 7). Staining of sections in a perpendicular direction of three different cell lines is shown in Figure 7C-E. The results of the SP-A staining on fetal lung tissue are shown in Figure 7A.

**Figure 8:**
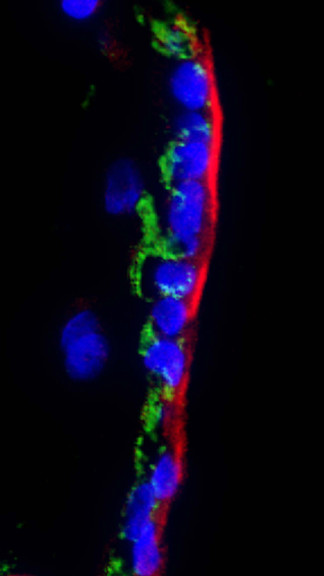
**Immunofluorescence double staining for SP-A and SP-C**. hESC (VUB03_DM1) were cultured with the ALI differentiation protocol for 20 days. Original magnification 600×. Nuclear DAPI staining in blue. Staining for SP-A (red) and SP-C (green) were combined. SP-A was found on one side of the cells, while SP-C can be seen in the cytoplasm. Similar findings were obtained for VUB07 (not shown).

To further specify the location of the proteins, a double staining was performed for SP-C and SP-A (Figure 8). A strongly positive SP-A staining pattern was detected on one side of the cell, while SP-C was detectable in the cytoplasm.

**Figure 9:**
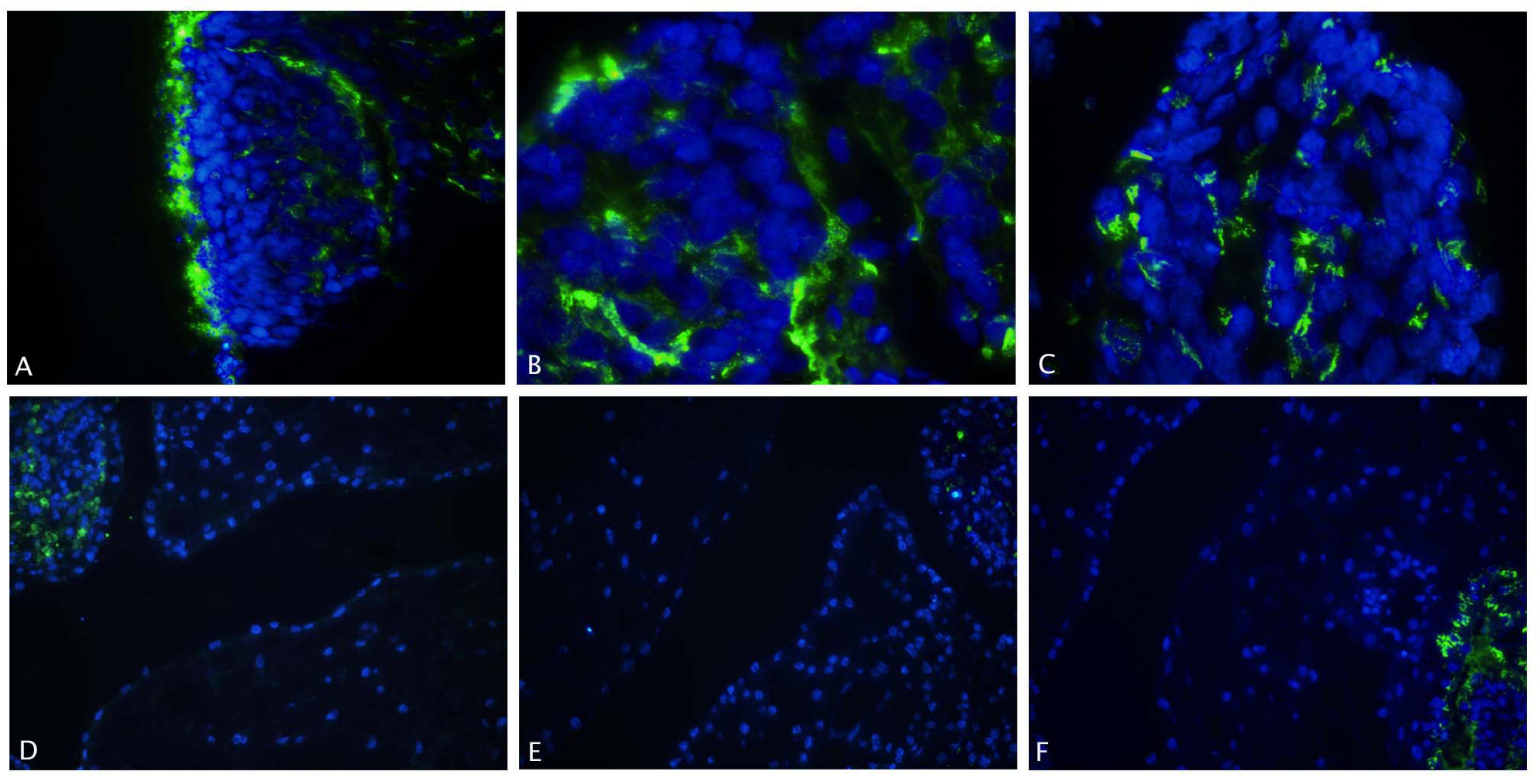
**Immunofluorescence staining for three stem cell markers: TRA1-60 (A,D), TRA1-81 (B,E) and SSEA4 (C,F)**. 4 μm thick sections of hESC grown with the ALI differentiation protocol for 20 days (D-F) were compared to sections of undifferentiated hESC control samples (A-C). Undifferentiated hESC showed extensive staining for all three markers. After 20 days of differentiation in ALI conditions only sporadic areas of staining could be observed. Representative results of VUB07 are shown. Original magnification: 200× (A, D-F) and 400× (B-C).

Staining for SSEA-4, TRA1-60 and TRA1-81, three human pluripotent stem cell markers, was performed to check for the presence of undifferentiated cells after 20 days of ALI culture in the ALI differentiation protocol (Figure 9). Undifferentiated hESC control samples were used as positive control for all three proteins. Small groups of undifferentiated cells could be detected scattered amongst the tissue.

### Expression of Vimentin by immunostaining for light microscopy

**Figure 10:**
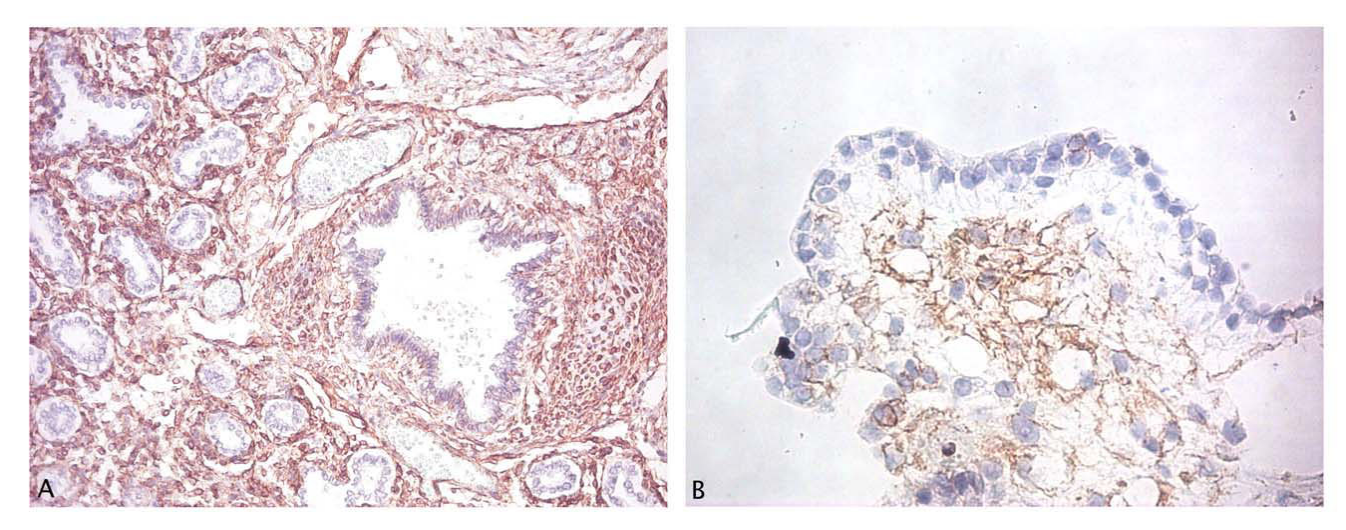
**Light microscopy images of Vimentin staining**. hESC (VUB03_DM1) were cultured with the ALI differentiation protocol for 20 days (B) and compared with staining in fetal lung tissue (A). Original magnification 100× (A), 200× (B). Staining for Vimentin was found in most cells except the epithelial-like cells. In fetal lung tissue (22 weeks old) staining can be seen in most cells except for bronchiolar epithelial cells.

A positive Vimentin staining was observed in most cells of fetal lung sections except for bronchiolar epithelial cells (Figure 10). After twenty days of ALI culture with the ALI differentiation protocol, brown spots could be seen in most of the differentiated hESC apart from epithelial-like cells at the border of the differentiated cell clumps.

### Quantitative determination of secreted Clara Cell 16 kD Protein by ELISA

**Figure 11:**
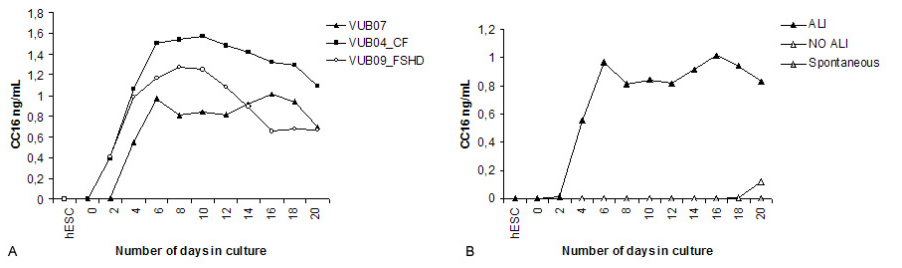
**Secreted CC16 protein was measured by ELISA**. CC16 secretion of three different hESC lines was compared. Secretion could be detected starting from two to four days after the induction of the ALI conditions. A similar secretion pattern over time could be measured in all three lines, with a peak in secretion level that is measured around day 8 followed by a decrease and stabilization further over time. (Figure 11A) CC16 secretion in samples from human embryonic stem cells (hESC) VUB07 cultured on porous membranes with the ALI differentiation protocol were compared to the NO ALI control samples and the "undifferentiated hESC control" (Figure 11B). All samples were measured in triplicate. No protein secretion could be detected in "No ALI control samples" till 20 days of culture. In the "spontaneous differentiation control" no secretion could be measured at any time. The minimum detectable concentration was <50 pg/mL.

The potential of Clara cells present in the ALI cultures to secrete CC16 was assessed by ELISA of collected cell culture supernatants. No CC16 protein was measured in the medium from undifferentiated hESC, while it could be measured as early as day two to four in ALI cultures. A steep incline was followed by a discreet decline further over time (Figure 11A).

The "NO ALI control" only gave a detectable secretion level after 20 days of ALI culture conditions, while no secretion could be measured at all in the "spontaneous differentiation control" samples. (Figure 11B)

## Discussion

This study describes a protocol to obtain lung epithelial-like tissue from human embryonic stem cells (hESC). The protocol relies on an air-liquid interface (ALI) system to mimic the conditions of an adult trachea and has been adapted from a protocol on murine ESC [10]. The specificity of the differentiation into lung epithelial cells was supported by expression data from quantitative real-time RT-PCR, immunohistochemistry and ELISA. Although most of the markers are not exclusively expressed in the lung, their cellular co-expression can be considered as sufficient to evaluate the generation of lung epithelial-like tissue.

Our protocol uses porous membranes to create an ALI to mimic the conditions of an adult trachea. It was previously shown on murine ESC [10] that these conditions support the differentiation into airway epithelial tissue. However, transfer of this protocol to hESC did not work. The protocol we use is a one step protocol (hESC plated directly on the porous membranes) instead of a two step protocol on murine ESC (plated on collagen before dissociation and transfer to the membrane). Furthermore the medium used in the two studies is different. Nevertheless, it should be mentioned that although our approach on human ESC is finally quite different from the protocol used on murine ESC, the results are similar. In both instances, the presence of *CC16 *mRNA early in the differentiation process (day 8 and day 10) was shown and there was evidence of differentiation into the main types of lung cells.

Although variation in mRNA expression level could be detected between the differentiating hESC lines, the expression pattern remained similar for all lines tested. The expression of *CC16*, a marker for Clara Cells, the nonciliated nonmucous cells lining the bronchioles of the lung, peaked significantly after ten days of differentiation with the ALI protocol. The same expression peak at day 10 was seen for *NKX2.1*, an early distal marker, while *SP-C *expression started to increase around day 20. This pattern resembles the lung development *in vivo*, where the level of *NKX2.1 *decreases with advancing age and the expression of the target genes such as *SP-A *and *SP-C *increases in association with differentiation of alveolar type II cells during the saccular-alveolar stage of lung development [17]. This was also confirmed by immunodetection of cytoplasmic SP-C and localization of SP-A at one side of the cells, corresponding with alveolar type II cells.

A significant increase in *β-tubulin IV *and *FOXJ1 *mRNA expression could be detected after twenty days of culture following the ALI differentiation protocol. *In vivo*, the FOXJ1 transcription factor functions in late-stage ciliogenesis of lung development and is expressed before the formation of ciliary 9+2 axonemes [8]. It therefore marks a lineage of cells distinct from those dependent upon *NKX2.1*. Both markers thus clearly show evidence for the differentiation of hESC into ciliated cells.

In addition, *Aquaporin 5*, a gene coding for a major water channel and in lung only expressed in alveolar type I cells, is also significantly increased after 20 days of culture, providing proof of cell maturation. The immunohistochemistry staining pattern for SP-A resembled the pattern seen in fetal lung tissue. This gene is expressed in lung alveolar type II cells and altered SP-A levels in Clara cells seem to play a role in diseases such as cystic fibrosis and respiratory distress syndrome [18-21]. In our study, we not only checked mRNA expression and protein localisation of several lung-specific genes, we also showed that one of these, CC16, is secreted by the cells and could be measured in the medium in our ALI culture conditions, but not in NO ALI control samples nor in spontaneous differentiated cell controls.

Vimentin is one of the mammalian intermediate filament proteins. It is expressed in cells of mesenchymal origin and is a feature of proliferating cells at the fetal stage. In our experiments *Vimentin *mRNA expression was measured both in our ALI cultures and in NO ALI control samples and both were compared to spontaneous differentiated control samples. No significant difference could be detected. Using immunostaining we showed the presence of Vimentin proteins in the ALI cultured tissue, ressembling Vimentin expression in fetal lung tissue.

The mRNA levels of the pluripotent stem cell markers POU5F1 and NANOG. showed a strong decrease, but were still detectable after twenty days of ALI culture. A decrease of these hESC-specific markers is generally observed upon differentiation. The RT-PCR analyses were confirmed by immunostaining for other stemness markers SSEA-4, TRA1-60 and TRA1-81; small groups of undifferentiated cells could be detected scattered amongst the tissue, indicating that only a small fraction of the cells remained undifferentiated after twenty days of ALI culture.

Other studies use small airway growth medium (SAGM), a commercial lung specific medium containing factors known to promote pneumocyte differentiation such as retinoic acid. One study on murine ESC describes a three-step protocol using Activin A in combination with low serum concentration to enhance the specification of the endodermal germ layer in embryoid bodies (EBs) and the final application of SAGM on plated EBs [9]. Results showed at least one cluster of SP-C expressing cells in the majority of the adhered embryoid bodies. Furthermore, the authors were able to measure the expression of several lung markers. Results of the same group [11] on human ESC were positive, but were only focused on alveolar type II cells. Here, hESC were allowed to differentiate spontaneously as EBs before plating, followed by changing the medium to SAGM ten days after plating. They have shown that hESC have the capacity to differentiate into lung-specific cells, expressing SP-C *in vitro*. Other research groups developed a transfection and culture procedure, which facilitates, via genetic selection the differentiation of hES cells into a relatively pure population (>99%) of alveolar type II cells [12].

A recent study on human amniotic fluid cells also shows their potential to differentiate into epithelial lung lineages [22]. For a review on these studies, we refer to a recent review by Rippon et al. [23].

In this study we demonstrate that hESC can differentiate into lung epithelial-like tissue without specific growth factors or embryoid body formation. The air-liquid interface on a porous membrane in combination with low serum is sufficient to prime the cells to form an airway epithelial-like tissue. It has been proven before that physical effects can direct stem cells [24].

Nevertheless, efforts will be made to further improve this novel culture protocol, trying to increase the number of differentiated cells or to guide the differentiation into particular cell types by adding certain growth factors to this system, such as fibroblast growth factors (FGF). These are important in the developing lung, and their addition to the culture medium may have an influence on the differentiation pattern [25].

The differentiation protocol was also tested on VUB04_CF, carrying one mutation in the *CFTR *gene. At first sight, no significant difference was noted between this line and other hESC lines. In the future, VUB04_CF and the newly derived VUB22_CF carrying the p.F508del mutation in both alleles can be used as model system to study differentiation effects on mRNA and protein levels.

It is remarkable that, although some chromosomal abnormalities could be found by array-based comparative genomic hybridization in the stem cell lines used in this study [14], results were comparable for all lines. These abnormalities seem to have no or only minor influence on the differentiation of hESC into lung epithelial-like tissue. It should be noted that our lines were checked by array-CGH, while most groups use G-banding obtained by Giemsa staining as a regular monitoring method, a technique that cannot detect small chromosomal aberrations.

To summarize, we developed a convenient protocol using ALI culture conditions that stimulates differentiation of human ESC into lung epithelial-like tissue. The combination of the mRNA expression results as well as results of protein expression and protein localisation showed the presence of the major cell types of lung epithelial tissue.

## Competing interests

The authors declare that they have no competing interests.

## Authors' contributions

LVH carried out the gene expression studies and cell culture and differentiation, performed the statistical analysis, participated in the ELISA tests and immunostainings and drafted the manuscript. GDB participated in the ELISA tests and immunostainings. IL was responsible for provision of study material and is supervisor. KS was responsible for financial support and participated in the design and coordination of the study. MDR conceived of the study, was responsible for financial support and participated in its design and coordination and in manuscript preparation. All authors read and approved the final manuscript.

## Supplementary Material

Additional file 1 **Characteristics of the hESC lines used**. The main characteristics of the cell lines, including the chromosomal abnormalities detected by array-CGH. Click here for file
