# Supplementary figures and images for: Generation of lung epithelial-like tissue from human embryonic stem cells
Source: Respir Res. 2009 Nov 5;10(1):105. doi: 10.1186/1465-9921-10-105 (PMC2777141; doi:10.1186/1465-9921-10-105)

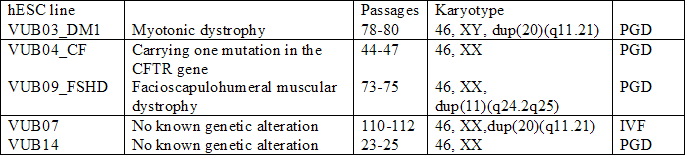

Supplement: Additional file 1 — Characteristics of the hESC lines used. The main characteristics of the cell lines, including the chromosomal abnormalities detected by array-CGH. [file 1465-9921-10-105-S1.png]
